# The impact of HCV co-infection status on healthcare-related utilization among people living with HIV in British Columbia, Canada: a retrospective cohort study

**DOI:** 10.1186/s12913-018-3119-5

**Published:** 2018-05-02

**Authors:** Huiting Ma, Conrado Franco Villalobos, Martin St-Jean, Oghenowede Eyawo, Miriam Ruth Lavergne, Lianping Ti, Mark W. Hull, Benita Yip, Lang Wu, Robert S. Hogg, Rolando Barrios, Jean A. Shoveller, Julio S. G. Montaner, Viviane D. Lima

**Affiliations:** 1 Department of Statistics, University of British Columbia, 3182 Earth Sciences Building, 2207 Main Mall, Vancouver, BC V6T 1Z4 Canada; 2 British Columbia Centre for Excellence in HIV/AIDS, 608 - 1081 Burrard Street, Vancouver, BC V6Z 1Y6 Canada; 3 Faculty of Health Sciences, Simon Fraser University, Blusson Hall, Room 10502, Burnaby, BC V5A 1S6 Canada; 4 British Columbia Centre for Excellence in HIV/AIDS, 667 - 1081 Burrard Street, Vancouver, BC V6Z 1Y6 Canada; 5 Department of Statistics, University of British Columbia, 3182 Earth Sciences Building room ESB 3126, 2207 Main Mall, Vancouver, BC V6T 1Z4 Canada; 6 School of Population & Public Health, University of British Columbia, 2206 East Mall, Rm 414, Vancouver, BC V6T 1Z3 Canada

**Keywords:** HIV, Hepatitis C virus, Healthcare utilization, Administrative data, Risk factors

## Abstract

**Background:**

The burden of HCV among those living with HIV remains a major public health challenge. We aimed to characterize trends in healthcare-related visits (HRV) of people living with HIV **(**PLW-HIV) and those living with HIV and HCV (PLW-HIV/HCV), in British Columbia (BC), and to identify risk factors associated with the highest HRV rates over time.

**Methods:**

Eligible individuals, recruited from the BC Seek and Treat for Optimal Prevention of HIV/AIDS population-based retrospective cohort (*N* = 3955), were ≥ 18 years old, first started combination antiretroviral therapy (ART) between 01/01/2000–31/12/2013, and were followed for ≥6 months until 31/12/2014. The main outcome was HRV rate. The main exposure was HIV/HCV co-infection status. We built a confounder non-linear mixed effects model, adjusting for several demographic and time-dependent factors.

**Results:**

HRV rates have decreased since 2000 in both groups. The overall age-sex standardized HRV rate (per person-year) among PLW-HIV and PLW-HIV/HCV was 21.11 (95% CI 20.96–21.25) and 41.69 (95% CI 41.51–41.88), respectively. The excess in HRV in the co-infected group was associated with late presentation for ART, history of injection drug use, sub-optimal ART adherence and a higher number of comorbidities. The adjusted HRV rate ratio for PLW-HIV/HCV in comparison to PLW-HIV was 1.18 (95% CI 1.13–1.24).

**Conclusions:**

Although HRV rates have decreased over time in both groups, PLW-HIV/HCV had 18% higher HRV than those only living with HIV. Our results highlight several modifiable risk factors that could be targeted as potential means to minimize the disease burden of this population and of the healthcare system.

**Electronic supplementary material:**

The online version of this article (10.1186/s12913-018-3119-5) contains supplementary material, which is available to authorized users.

## Background

In high-income settings, HIV infection has become a chronic manageable condition [1]. Overall, HIV/AIDS associated morbidity and mortality has decreased to unprecedented levels, largely due to the widespread use of combination antiretroviral therapy (ART) [2]. People living with HIV/AIDS (PLW-HIV) now have life expectancies comparable to those observed in the general population, although variability between sub-groups remain [3]. Despite successful treatment-mediated viral suppression, premature morbidity and mortality due to non-AIDS related infectious and non-infectious comorbidities are increasingly prevalent, raising new challenges for healthcare providers and health systems [4].

Globally, the hepatitis C virus (HCV) has become one of the most prevalent co-infection among PLW-HIV [5]. Indeed, HCV mortality has surpassed that of all other reportable infectious diseases together, including AIDS and tuberculosis, in the United States [6]. According to a recent meta-analysis, the prevalence of both HIV and HCV is the highest (82%) among people who inject drugs (PWID) [5]. In Canada, similar to the United States, 20% to 30% of PLW-HIV are also living with HCV, while the prevalence of both viruses among PWID ranges between 50% and 90% [7–9]. In addition, the presence of both viruses has been shown to increase the risk for clinical progression of HIV as well as premature mortality (despite ART), and accelerated progression of HCV-associated liver diseases [10, 11].

Enhanced immunosenescence, resulting from HIV infection via persistent inflammatory activity and immune activation, is associated with increased morbidity and mortality [12, 13]. It is becoming increasingly evident that HCV infection also contributes to systemic immunosenescence [14–16]. Of note, HCV-related disease burden is not restricted to the liver; it extends to several conditions (immune-mediated or activated by chronic inflammation) impacting extrahepatic organs/tissues [17]. Additionally, life-style factors, socio-economic constraints, and inadequate engagement in care tend to exacerbate other comorbid conditions, further complicating clinical outcomes among some subgroups of PLW-HIV, particularly those co-infected with HCV [18, 19].

To date, a large number of people living with HIV and HCV (PLW-HIV/HCV) have no or limited access to HCV treatment. However, very recently, the healthcare landscape in British Columbia (BC), Canada, has been rapidly transforming with the advent of highly efficacious and tolerated HCV direct-acting antiviral therapies, resulting in a steady increase in the number of individuals accessing this life-saving therapy [20, 21]. Still, there are barriers for accessing healthcare services and programs for those afflicted by these diseases that need to be identified, particularly among those who are marginalized and vulnerable in the population [20].

Thus, the main objective of this study was to characterize the trends in healthcare-related visits (HRV) of PLW-HIV and PLW-HIV/HCV, in BC. Additionally, we aimed at identifying modifiable risk factors, associated with the highest HRV rates over time that could be targeted as potential means to minimize the disease burden of this population and on the healthcare system.

## Methods

### Study setting

The province of BC established the BC Centre for Excellence in HIV/AIDS Drug Treatment Program (DTP) in 1992; it has since been responsible for the distribution of antiretrovirals [22]. The DTP, funded by the provincial government, provides HIV medical care and laboratory monitoring (e.g., CD4 cell counts and viral load) for all diagnosed PLWH residing in BC at no cost, in accordance with BC’s HIV therapeutic guidelines, which have largely remained consistent with those of the International Antiviral Society-USA since 1996 [23, 24].

### Study design and data

This retrospective study was carried out using data from the British Columbia Seek and Treat for Optimal Prevention of HIV/AIDS (STOP HIV/AIDS) population-based cohort, which is derived from various linkages among provincial databases [22, 25–30]. This cohort is briefly described in the Additional file 1. Our inclusion criteria for individuals were as follows: (i) ART-naïve individuals aged ≥18 years, (ii) enrolled in the (DTP) between January 1, 2000 and December 31, 2013, (iii) initiated ART consisting of two nucleoside/nucleotide reverse transcriptase inhibitors (NRTIs) as backbone, plus either a non-nucleoside reverse transcriptase inhibitor (NNRTI) or a ritonavir-boosted protease inhibitor (bPI), (iv) had a CD4 count and a viral load measurement within 6 months of ART treatment initiation, and (v) had at least 6 months of follow-up. Note that we decided to exclude other initial ART regimens due to the small number of individuals who have initiated on them. Eligible individuals were followed until December 31, 2014, the last contact date (i.e., the last available laboratory test date, the last filled ART prescription refill date or the date of last encounter with the healthcare system identified in any of the STOP HIV/AIDS databases), or the date of death (all-causes).

All viral load tests and the majority of CD4 cell count tests were performed by the St. Paul’s Hospital laboratories in Vancouver, BC, and were subsequently transferred to the DTP via electronic linkage. CD4 cell count tests completed at other laboratories throughout BC were manually entered into the DTP; altogether resulting in approximately 85% data capture of all CD4 cell count tests done in the province. For analytical purposes, all viral load measurements were transformed to range from < 50 (coded as 49) to > 100,000 (coded as 100,010) copies/mL. This process was necessary to account for advances in testing methodology, as previously described elsewhere [31]. CD4 cell counts were measured by flow cytometry (Beckman Coulter, Inc., Mississauga, Ontario).

### Main outcome

The main outcome was the rate of HRV per individual for every 6-month interval. HRV was calculated based on records from the Medical Services Plan (MSP) billing provincial database linked to STOP HIV/AIDS cohort. MSP captures HIV and non-HIV-related inpatient and outpatient services provided by physicians and supplementary health care practitioners, as well as diagnostic procedures. The unit of analysis for the crude HRV rate was person-year, which was calculated by dividing the number of HRV by the number of person-years of follow-up in each calendar year. The corresponding 95% confidence intervals (CI) for these rates were based on the Fisher’s exact test [32]. Note that we also presented the age-sex standardized HRV rates for the overall follow-up utilizing BC’s population estimates as the reference [33].

The number of HRV was derived from the MSP physician billing records, which contain the date, type and location of service, physician number, practitioner speciality and costs. Since multiple MSP database records could be associated with one unique HRV, a record was considered to be a unique HRV if it satisfied one of the following conditions:

i. If the service date, which is the date on which the service was rendered by a practitioner, was different; or
ii. If the speciality number, which is a number assigned to identify the practitioner’s specialty, was different; or
iii. If the location of the service was different.

Once the rules outlined above were adopted, we identified the unique HRV for these individuals. Subsequently, we calculated the number of HRV in each 6-month interval for all individuals. Visits were classified as general practitioners, other healthcare practitioners, and laboratory services.

### Main exposure variable

The main exposure variable of interest was HIV/HCV co-infection status, derived from the DTP database, which indicates whether PLW-HIV has ever had evidence of HCV infection (i.e., a HCV antibody positive or HCV RNA detected, as indicated by laboratory result data or physician reported status). Note that upon successful HCV treatment, individuals can become re-infected with HCV. Thus, we were only able to incorporate HCV ever status for these individuals.

### Potential confounders

The confounders measured at ART initiation included: sex (male, female), risk for HIV acquisition (gay, bisexual and other men who have sex with men (MSM), PWID, MSM/PWID, Other, Unknown), initial ART regimen (NNRTI, bPI) and period of ART initiation (2000–2003, 2004–2007, 2008–2011, 2012–2013). Several time-varying confounders, measured every year, were considered in this study, including: age (< 30, 30–39, 40–49, ≥50 years), CD4 cell count (< 50, 50–199, 200–349, ≥350 cells/mm^3^), viral load (log_10_ transformed), adherence level (< 40%, 40–79%, 80–94%, ≥95%), cumulative number of comorbid diseases (0, 1, 2, ≥3) and person-years of follow-up time. Adherence level was determined on the basis of a validated measure assessing refill compliance, which was calculated by dividing the amount of days of dispensed ART medication by the amount of days of study follow-up, for each period (presented as percentage). ART adherence calculations were derived from distinct regimen exposures for each individual [34]. PWID included individuals with past and current exposure to injection drug use. Individual comorbidities were derived from the Charlson Comorbidity Index (Additional file 1: Table S1) [35]. These included 16 conditions, other than HIV/AIDS (e.g., renal, liver, heart and lung diseases and cancer). These conditions were identified using International Classification of Diseases (Ninth and Tenth Revisions, Clinical Modification) diagnosis codes obtained from the STOP HIV/AIDS cohort databases based on a validated case-finding algorithm [36].

### Statistical analysis

Categorical variables were compared using the Fisher’s exact test or the *X*^2^ test, and continuous variables were compared using the Kruskal-Wallis test [37]. Based on exploratory data analyses to determine the best distribution for modeling HRV rates, non-linear mixed effects models were used assuming a Poisson distribution, the person-years of follow-up time as the offset, a log link function, a random intercept term and an autoregressive of order one working correlation matrix [38]. We have chosen these models since they are flexible in taking into account the inter- and intra-individual sources of variation, they can handle imbalanced longitudinal data, and zero-inflated models did not show any gain over the final fitted model [38, 39]. Potential confounders were selected for inclusion in the final model using a backward-selection approach, published by our group based on the work by Maldonado and Greenland [40], that considered the magnitude of change in the coefficient of the HIV/HCV co-infection status variable. Specifically, starting with a fixed model, which considered all available variables, potential confounders were dropped one at a time, using the relative change in the coefficient for the variable related to the HIV/HCV co-infection status as a criterion, until the maximum change from the full model exceeded 5% [41]. Note that in the multivariable model, we did not adjust for risk for HIV acquisition given its high collinearity with the main study exposure. All analyses were performed using either R© version 3.3.2 (The R foundation for statistical computing, Vienna, Austria) or SAS version 9.4 (SAS, Cary, North Carolina, USA).

## Results

### Cohort characteristics

Overall, 4217 ART-naïve adults with a total of 615,776 HRV were initially eligible to participate in this study. Among these individuals, 81% were males, 67% were aged between 30 and 49 years, 72% were either MSM, PWID or both, 40% started ART between 2008 and 2011, 76% initiated ART with a CD4 cell count < 350 cells/mm^3^ and had a median viral load 4.90 log_10_ copies/mL (25th–75th percentile (Q1-Q3): 4.38–5.00 log_10_ copies/mL), 52% started on a bPI-based ART, 78% had adherence ≥95% during the first six months on ART, and 34% had no comorbidities while 14% had 3 or more. The median follow-up time was 4.99 (Q1-Q3: 2.50–7.98) years, in which the median number of HRV was 97 (Q1-Q3: 48–187) (Table 1). This cohort comprised of 2333 (55%) PLW-HIV, 1622 (39%) PLW-HIV/HCV, and 262 (6%) whose HCV status was unknown. The distribution of study variables among those included in this analysis (*N* = 3955) was very similar to the original 4217 individuals described above. For the purposes of this study, those with unknown HCV status, who contributed 25,393 HRV (4% of total visits), were excluded from the subsequent analyses. As noted in Table 1, those excluded were more likely to have risk for HIV acquisition other than MSM or PWID, a lower number of HRV and a slight shorter follow-up time.

**Table 1:** Study population characteristics by inclusion status

Variables	Overall	Included	Excluded	*P*-value
	*N* = 4217	*N* = 3955	*N* = 262	
Sex, n(%)				
Female	805 (19)	735 (91)	70 (9)	0.0016
Male	3412 (81)	3220 (94)	192 (6)	
Status				
HIV mono-infected	2333 (55)	2333 (100)	0 (0)	NA
HIV/HCV co-infected	1622 (39)	1622 (100)	0 (0)	
Unknown	262 (6)	0 (0)	262 (100)	
Age at ART initiation (years), n (%)				
< 30	516 (12)	481 (93)	35 (7)	0.1724
30–39	1294 (31)	1216 (94)	78 (6)	
40–49	1523 (36)	1441 (95)	82 (5)	
≥ 50	884 (21)	817 (92)	67 (8)	
Risk, n(%)				
MSM	1376 (33)	1294 (94)	82 (6)	< 0.0001
PWID	1335 (32)	1298 (97)	37 (3)	
MSM/ PWID	314 (7)	309 (98)	5 (2)	
Other	652 (15)	581 (89)	71 (11)	
Unknown	540 (13)	473 (88)	67 (12)	
ART era, n(%)				
2000–2003	859 (20)	797 (93)	62 (7)	0.1037
2004–2007	1214 (29)	1153 (95)	61 (5)	
2008–2011	1704 (40)	1599 (94)	105 (6)	
2012–2013	440 (10)	406 (92)	34 (8)	
Baseline CD4 cell count (cells/mm^3^), n (%)				
< 50	498 (12)	474 (95)	24 (5)	0.4136
50–199	1366 (32)	1285 (94)	81 (6)	
200–349	1329 (32)	1238 (93)	91 (7)	
≥ 350	1024 (24)	958 (94)	66 (6)	
ART Adherence (first six months), n (%)				
≥ 95%	3291 (78)	3084 (94)	207 (6)	0.7563
80–94%	218 (5)	208 (95)	10 (5)	
40–79%	474 (11)	445 (94)	29 (6)	
< 40%	234 (6)	218 (93)	16 (7)	
Number of comorbidities at baseline, n(%)				
0	1451 (34)	1357 (94)	94 (6)	0.8122
1	1397 (33)	1307 (94)	90 (6)	
2	768 (18)	725 (94)	43 (6)	
≥3	601 (14)	566 (94)	35 (6)	
Initial ART regimen, n(%)				
NNRTI	2033 (48)	1895 (93)	138 (7)	0.1531
bPI	2184 (52)	2060 (94)	124 (6)	
Total healthcare-related visits, median (Q1-Q3)	97 (48–187)	99 (49–191)	71 (31–125)	< 0.0001
Baseline viral load (log_10_ copies/mL), median (Q1-Q3)	4.90 (4.38–5.00)	4.90 (4.38–5.00)	4.85 (4.28–5.00)	0.4339
Follow-up time (years), median (Q1-Q3)	4.99 (2.50–7.98)	4.99 (2.81–8.00)	3.50 (1.76–5.99)	< 0.0001

Q1-Q3: 25th - 75th percentiles; MSM: Gay, bisexual and other men who have sex with men; PWID: people who have ever injected drugs; ART: combination antiretroviral therapy; NNRTI: non-nucleoside reverse transcriptase inhibitor; bPI: ritonavir-boosted protease inhibitor; NA: not applicable. Note that Overall column shows column percent, while Included/Excluded columns show row percent

### Characteristics by HIV/HCV co-infection status

Bivariable analysis exploring associations between study characteristics and HCV co-infection status (shown in Table 2) revealed that PLW-HIV and PLW-HIV/HCV differed significantly in all study characteristics, except for initial ART regimen. PLW-HIV/HCV were more likely to be younger, female, PWID, initiate ART prior to 2008, have a lower CD4 cell count and viral load measurement at baseline, and maintain adherence < 40% during follow-up. Additionally, these individuals were also more likely to present with and develop a higher number of comorbidities compared to PLW-HIV. The number of PLW-HIV/HCV with moderate or severe liver disease increased substantially during follow-up (75 (5%) to 124 (8%); *p*-value 0.0004). Apart from liver disease, chronic pulmonary disease and cancer (all causes) were the most prevalent comorbidities (results not shown).

**Table 2:** Study population characteristics by hepatitis C (HCV) co-infection status

Variables	HIV mono-infected	HIV/HCV co-infected	*P*-value
	*N* = 2333	*N* = 1622	
Sex, n(%)			
Female	269 (37)	466 (63)	< 0.0001
Male	2064 (64)	1156 (36)	
Age at ART initiation (years), n(%)			
< 30	304 (63)	177 (37)	< 0.0001
30–39	726 (60)	490 (40)	
40–49	780 (54%)	661 (46%)	
≥ 50	523 (64%)	294 (36%)	
Risk, n(%)			
MSM	1144 (88)	150 (12)	< 0.0001
IDU	122 (9)	1176 (91)	
MSM/IDU	122 (39)	187 (61)	
Other	514 (88)	67 (12)	
Unknown	431 (91)	42 (9)	
ART era, n(%)			
2000–2003	403 (51)	394 (49)	< 0.0001
2004–2007	628 (54)	525 (46)	
2008–2011	1001 (63)	598 (37)	
2012–2013	301 (74)	105 (26)	
Baseline CD4 cell count (cells/mm^3^), n(%)			
< 50	276 (58)	198 (42)	< 0.0001
50–199	640 (50)	645 (50)	
200–349	746 (60)	492 (40)	
≥ 350	671 (70)	287 (30)	
Last CD4 cell count (cells/mm^3^), n(%)			
< 50	40 (33)	81 (67)	< 0.0001
50–199	101 (31)	228 (69)	
200–349	232 (46)	277 (54)	
≥ 350	1732 (67)	864 (33)	
Unknown	228 (57)	172 (43)	
ART Adherence (first six months), n(%)			
≥ 95%	1987 (64)	1097 (36)	< 0.0001
80–94%	100 (48)	108 (52)	
40–79%	182 (41)	263 (59)	
< 40%	64 (29)	154 (71)	
ART Adherence (last six months), n(%)			
≥ 95%	1821 (65)	960 (35)	< 0.0001
80–94%	122 (47)	140 (53)	
40–79%	224 (45)	277 (55)	
< 40%	166 (40)	245 (60)	
Number of comorbidities at baseline, n(%)			
0	1067 (79)	290 (21)	< 0.0001
1	745 (57)	562 (43)	
2	331 (46)	394 (54)	
≥3	190 (34)	376 (66)	
Number of comorbidities at the end of follow-up, n(%)			
0	961 (82)	215 (18)	<0.0001
1	687 (62)	421 (38)	
2	333 (47)	382 (53)	
≥3	352 (37)	604 (63)	
Initial ART regimen, n(%)			
NNRTI	1098 (58)	797 (42)	0.2109
bPI	1235 (60)	825 (40)	
Total healthcare-related visits, median (Q1-Q3)	83 (43–150)	143 (68–278)	< 0.0001
Baseline viral load (log_10_ copies/mL), median (Q1-Q3)	4.92 (4.42–5.00)	4.87 (4.35–5.00)	0.0048
Last viral load (log_10_ copies/mL), median (Q1-Q3)	1.69 (1.69–1.69)	1.69 (1.69–1.98)	< 0.0001
Follow-up time (years), median (Q1-Q3)	4.95 (2.50–7.91)	5.30 (2.99–8.31)	0.0038

Q1-Q3: 25th - 75th percentiles; MSM: Gay, bisexual and other men who have sex with men; PWID: people who have ever injected drugs; ART: combination antiretroviral therapy; NNRTI: non-nucleoside reverse transcriptase inhibitor; bPI: ritonavir-boosted protease inhibitor

### Trends in healthcare-related visits

**Fig. 1:**
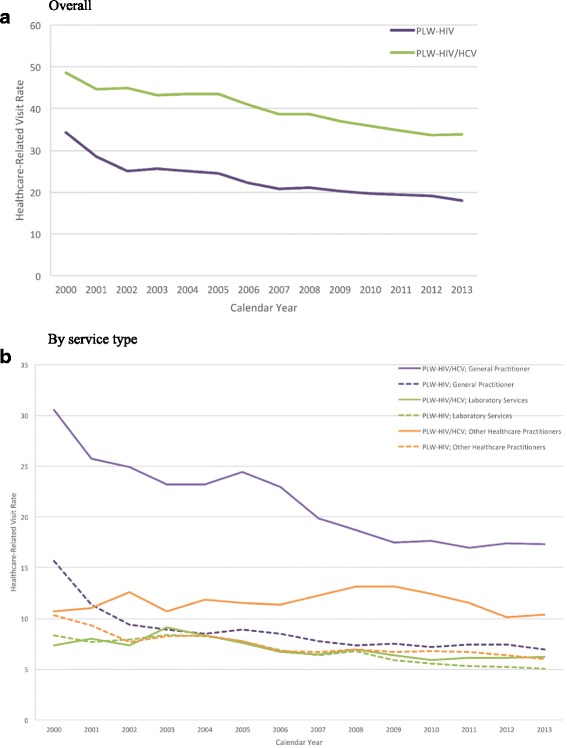
Crude rate of healthcare-related visits (per person-year) for PLW-HIV and PLW-HIV/HCV, from 2000 to 2013. Panel **a**, corresponds to the overall healthcare-related visit rate. Panel **b** corresponds to the healthcare-related visit rate stratified by service type

The overall age-sex standardized HRV rate for PLW-HIV and PLW-HIV/HCV was 21.11 per person-year (95% CI 20.96–21.25) and 41.69 per person-year (95% CI 41.51–41.88), respectively. For PLW-HIV/HCV, visits were mainly related to methadone or buprenorphine/naloxone treatment, and not to the same extent, to laboratory tests done to monitor liver and kidney function and the hematology profile. For PLW-HIV, most visits were concerned with the latter. As demonstrated in Fig. 1a, the annual crude HRV rates in both groups have been steadily decreasing from 2000 to 2013. The decrease in HRV rates was more prominent for PLW-HIV (34.28 per person-year to 17.98 per person-year; 48% decrease; *p*-value < 0.0001) compared to PLW-HIV/HCV (48.57 per person-year to 33.92 per person-year; 30% decrease; p-value < 0.0001). The trends in HRV rates stratified by HIV/HCV co-infection status and by the type of HRV are illustrated in Fig. 1b. As shown, except for HRV rates related to laboratory services, PLW-HIV/HCV consistently maintained higher rates, especially those related to general practitioner visits (> 2 times higher). Note that the rates for other healthcare practitioners (which includes visits to specialists), although much lower than the HRV rates for general practitioner visits, they have been more stable over time and the difference between these groups was not as pronounced. More detailed information on these trends can be found in Additional file 1: Table S2. The result of the multivariable confounder model showed that the adjusted HRV rate ratio for PLW-HIV/HCV in comparison to PLW-HIV was 1.18 (95% CI 1.13–1.24), after controlling for sex, age, ART era, time-varying CD4, adherence to ART, viral load and the number of comorbidities (Table 3).

**Table 3:** Results from the multivariable confounder model

Variables	Rate Ratio (95% Confidence Interval)	
	Unadjusted Model	Adjusted Model
Status		
HIV mono-infected	1 (−)	1 (−)
HIV/HCV co-infected	1.65 (1.57–1.73)	1.18 (1.13–1.24)
Sex		
Male	1 (−)	1 (−)
Female	1.45 (1.36–1.54)	1.19 (1.13–1.25)
Age at ART initiation (years)		
< 30	1 (−)	1 (−)
30–39	1.08 (1.07–1.10)	Not selected
40–49	1.23 (1.21–1.25)	
≥ 50	1.47 (1.43–1.50)	
ART era		
2000–2003	1 (−)	1 (−)
2004–2007	0.84 (0.77–0.93)	Not selected
2008–2011	0.82 (0.76–0.87)	
2012–2013	0.94 (0.87–1.00)	
CD4 cell count (cells/mm^3^) (time-varying)		
< 50	2.29 (2.25–2.32)	2.27 (2.24–2.31)
50–199	1.45 (1.44–1.47)	1.44 (1.43–1.46)
200–349	1.22 (1.21–1.23)	1.22 (1.21–1.23)
≥ 350	1 (−)	1 (−)
Unknown	1.00 (0.99–1.01)	1.00 (0.98–1.01)
ART Adherence (time-varying)		
≥ 95%	1 (−)	1 (−)
80–94%	1.19 (1.17–1.20)	Not selected
40–79%	1.20 (1.19–1.22)	
< 40%	1.12 (1.11–1.13)	
Number of comorbidities (time-varying)		
0	1 (−)	1 (−)
1	1.39 (1.32–1.47)	1.30 (1.23–1.37)
2	1.87 (1.76–1.99)	1.64 (1.54–1.75)
≥3	2.82 (2.67–2.99)	2.34 (2.21–2.48)
Initial ART regimen		
NNRTI	1 (−)	1 (−)
bPI	1.12 (1.07–1.18)	Not selected
Viral load (log_10_ copies/mL) (time-varying)	1.13 (1.13–1.13)	Not selected

ART: combination antiretroviral therapy; NNRTI: non-nucleoside reverse transcriptase inhibitor; bPI: ritonavir-boosted protease inhibitor. Note that in the multivariable model we did not adjust for risk for HIV acquisition given its high collinearity with the main study exposure. Not selected means that the variable was not a confounder in the model

## Discussion

This population-based cohort study adds to the growing body of evidence indicating that PLW-HIV/HCV incur significantly greater HRV rates relative to those only living with HIV [42–44]. It is worth noting that although PLW-HIV/HCV experienced an 18% higher rate relative to PLW-HIV, we observed a decrease in HRV rates over time among both groups, even after controlling for several confounders including disease severity and the cohort effect. Apart from HCV infection, the excess in HRV rates among PLW-HIV/HCV were at least partially attributable to the fact that these individuals had a history of injection drug use, presented later for HIV treatment, had sub-optimal adherence to ART and had higher prevalence of comorbidities.

To understand the reason behind the decreasing rates over time, the reader should be aware that the study period encompasses three phases of BC’s response to HIV/AIDS: the harm reduction and health service scale-up phase (2000–2005); the early Treatment as Prevention phase (2006–2009); and the STOP HIV/AIDS phase (2010-present), during which BC’s HIV therapeutic guidelines recommended ART treatment for all adults with HIV infection, regardless CD4 count [45, 46]. Throughout these phases, various HIV care initiatives have been implemented and may have attenuated the healthcare-related utilization of this population. Namely, the evolving deployment of biomedical and health service interventions (e.g., the development of improved antiretroviral drugs, substance use treatment, and medication adherence support) and structural interventions (e.g., legal and policy), which have been comprehensive described elsewhere [45].

We should also note that both groups of individuals were linked to HIV care and receiving treatment. Most likely, if people only living with HCV were included in this analysis, we would see that these trends, in this same period, did not decrease given that, in BC, most of these individuals are not fully engaged into care. In addition, given the recent approval for use of safer, more tolerable and efficacious interferon-free direct acting antivirals-based HCV therapy, going forward, these trends will likely change, particularly for specialist-related visits as they will be the ones mainly prescribing these medications and following these individuals. Thus, there is a need for continued monitoring and evaluation of HRV among PLW-HIV/HCV, especially since in 2017, the BC Ministry of Health has announced a province-wide expansion of HCV treatment to all of those living with HCV starting in March 2018 [21].

The persisting disparity in HRV rates observed among PLW-HIV and PLW-HIV/HCV clearly indicates that there is a critical need for interventions that may attenuate the risk of requiring higher resource use for care among those living with both viruses. Additionally, any successful strategy to attenuate the utilization of PLW-HIV/HCV will require significant levels of treatment uptake and adherence, especially among PWID (including PLW-HIV/HCV). Our data also suggest that addressing the underlying substance use disorder may be beneficial in this regard. Furthermore, doing so would also contribute to preventing HCV reinfection, which would greatly enhance the individual and societal impact of interferon-free direct acting antivirals-based HCV therapy [47–49]. On that note, several clinical models have proved to be successful in this regard by combining services aimed to address viral hepatitis and HIV, substance use detoxification, opioid substitution, and primary care in low threshold environment, coupled with comprehensive and integrated multidisciplinary teams of health care professionals including treaters, nurses, substance use and behavioral health service providers, as well as other social support services [48]. In 2016, the BC Centre for Excellence in HIV/AIDS launched a province-wide monitoring and evaluation strategy, which will address the health needs of those living with or at risk of HCV infection, including those also living with HIV [50, 51]. The key aims of this program include the normalization of HCV testing, especially among those at higher risk; support to facilitate access to HCV and substance use treatment; extensive deployment of harm reduction strategies; and strengthening of educational programs to treat and care for this population.

The implications of HIV/HCV co-infection in the context of a rapidly expanding population of aging PLW-HIV are important, particularly at a time when meeting health demands in BC, and in other high-resource settings, is becoming exceedingly challenging amid fiscal constraints [52]. As PLW-HIV live longer and non-AIDS-related comorbidities continue to rise, the impact of HIV/HCV co-infection will be increasingly relevant, both from a clinical perspective and a health systems perspective. Chronic HCV infection among PLW-HIV may have contributed to the exacerbation of progressive immunosenescence, and it may be associated with premature morbidity and mortality manifested by the development of multiple comorbidities (as observed in this study) [16, 53], further contributing to increased financial strain on healthcare systems.

The findings of the present study should be interpreted in light of several limitations. First, HIV**/**HCV co-infection status was assigned based on ever having recorded a positive HCV antibody test or detected HCV RNA. Thus, it is unknown whether these individuals had active HCV infection during the study period. Second, several factors such as rates of spontaneous viral clearance, HCV treatment and re-infection (among those successfully treated) were unknown for this cohort, thus limiting our ability to identify and adjust for these factors in the model. Third, therapy for opioid dependence was not considered, but may have impacted HRV among PLW-HIV/HCV with active injection drug use. Fourth, although healthcare administrative data are an important source of information for evidence-based clinical and policy decision-making as well as medical research, we should note that these data are susceptible to inaccurate or incomplete coding, potentially leading to missing or misclassified HRV in both groups. Finally, while this study examined HRV exclusively, there are other forms of healthcare utilization not accounted for in these analyses (e.g., addiction support services).

## Conclusions

In conclusion, in this retrospective study, we found that although HRV rates have been decreasing steadily over time, PLW-HIV/HCV consistently maintained higher HVR rates relative to PLW-HIV. Our results highlight several modifiable risk factors (i.e., late presentation for ART, injection drug use, sub-optimal ART adherence and comorbidities) that could be targeted as potential means to minimize the disease burden of this population and on the healthcare system.

## Additional file

Additional file 1: Supplementary information detailing the data linkage within the STOP HIV/AIDS cohort, the ascertainment of comorbidities based on the Charlson Comorbidity Index, and the number of healthcare-related visits recorded. (DOCX 35 kb)

## Abbreviations

ART: Combination antiretroviral therapy
BC: British Columbia
bPI: Ritonavir-boosted protease inhibitor
CI: Confidence Interval
HCV: Hepatitis C virus
HRV: Healthcare-related visits
MSM: Men who have sex with men
MSP: Medical Services Plan
NNRTI: Non-nucleoside reverse transcriptase inhibitor
NRTI: Nucleoside/nucleotide reverse transcriptase inhibitor
PLW-HIV: People living with HIV/AIDS
PLW-HIV/HCV: People living with HIV and HCV
PWID: People who inject drugs
STOP HIV/AIDS: Seek and Treat for Optimal Prevention of HIV/AIDS
