# Supplementary material for: The impact of HCV co-infection status on healthcare-related utilization among people living with HIV in British Columbia, Canada: a retrospective cohort study
Source: BMC Health Serv Res. 2018 May 2;18:319. doi: 10.1186/s12913-018-3119-5 (PMC5932856; doi:10.1186/s12913-018-3119-5)
Supplement: Supplementary file 1 — Supplementary information detailing the data linkage within the STOP HIV/AIDS cohort, the ascertainment of comorbidities based on the Charlson Comorbidity Index, and the number of healthcare-related visits recorded. (DOCX 35 kb) [file 12913_2018_3119_MOESM1_ESM.docx]

**The impact of HCV co-infection status on healthcare-related utilization among people iving with HIV in British Columbia, Canada: A retrospective cohort study**

**Additional file 1**

This study was carried out using data from the British Columbia Seek and Treat for Optimal Prevention of HIV/AIDS (STOP HIV/AIDS) population-based cohort, which is derived from various linkages between provincial administrative databases.

**Data Steward: British Columbia Centre for Disease Control** (1, 2)

1. Provincial HIV/AIDS Surveillance Database: a surveillance database that collates all HIV laboratory testing, new HIV diagnosis and occurrence of AIDS-defining illnesses data;

**Data Steward: British Columbia Centre for Excellence in HIV/AIDS** (3)

1. Drug Treatment and Laboratory Databases, which captures all antiretroviral dispensing data, plasma viral load testing, drug resistance testing, occurrence of AIDS-defining illnesses, approximately 85% of CD4 cell count measurements, and key patient demographic information;

**Data Steward: British Columbia Ministry of Health** (4)

1. The Medical Services Plan (MSP) billing database, which captures HIV and non-HIV-related inpatient and outpatient services provided by physicians and supplementary health care practitioners, as well as diagnostic procedures. This database also contains cost associated with claims paid through fee-for-service and the Alternative Payment Program;
2. Home and Community Care database, which captures a variety of services including hospice and home nursing care, adult day services, assisted living, respite care, residential and convalescent care
3. Mental Health Services database, which captures utilization of mental health services including fee-for-service, institutional care, community clinics and acute care.
4. The PharmaNet database is a real-time system, which captures all prescriptions for drugs and medical supplies dispensed from community pharmacies in BC as well as prescriptions dispensed from hospital outpatient pharmacies use at home. Note that this database does not capture antiretroviral dispensing data;
5. The Client Roster or Consolidation File, which captures individual demographic and geographic data. This database is also used to construct population denominators.

**Data Steward: Canadian Institute for Health Information** (5)

1. The Discharge Abstract Database (DAD), which captures all discharges, transfers and deaths of in-patients and day surgery patients from acute care hospitals across BC;

**Data Steward: British Columbia Vital Statistics Agency** (6)

1. The Vital Statistics database, which records death information of all BC’s residents.

**Table 1.** Individual comorbidities derived from the Charlson Comorbidity Index, based on the International Classification of Diseases (Ninth and Tenth Revisions, Clinical Modification) diagnosis codes (7).

| **Comorbidities** | **International Classification of Diseases, Ninth Revision, Clinical Modification, (ICD-9-CM) Codes** | **International Classification of Diseases, Tenth Revision, Clinical Modification, (ICD-10-CM) Codes** |
| --- | --- | --- |
| Myocardial infarction | 410,412 | I21, I22, I252 |
| Congestive heart failure | 398,402,425,428 | I43, I50, I099, I110, I130, I132, I1255, I420, I425, I426 |
| Peripheral vascular disease | 440,441,443,447,557 | I70, I71, I731, I738, I739, I771, I790, I792, K551, K558, K559, Z958, Z959 |
| Cerebrovascular disease | 430,431,432,433,434,435,436,437,438 | G45, G46, I60, I61, I62, I63, I64, I65, I66, I67, I68, I69, H340 |
| Dementia | 290,294,331 | F00, F01, F02, F03, G30, F051, G311 |
| Chronic pulmonary disease | 490, 491, 492, 493, 494, 495, 496, 500, 501, 502, 503, 504, 505 | J40, J41, J42, J43, J33, J45, J46, J47, J60, J61, J62, J63, J64, J65, J66, J67, I278, I279, J684, J701, J703 |
| Connective tissue disease/  rheumatic disease | 446, 710, 714, 725 | M05, M32, M33, M34, M06, M315, M351, M353, M360 |
| Peptic ulcer disease | 531, 532, 533, 534 | K25, K26, K27, K28 |
| Mild liver disease | 570, 571, 573 | B18, K73, K74, K7000, K701, K702, K703, K709, K717, K713, K714, K715, K760, K762, K764m K768, K769, Z944 |
| Moderate to severe liver disease | 465, 572 | K711, K729, K765, K766, K767, I850, I859, I859, I864, I982, K704, |
| Diabetes without complications | 250 | E100, E101, E106, E108, E109, E110, E111, E116, E118, E119, E120, E121, E126, E128, E129, E130, E131, E136, E138, E139, E140, E141, E146, E148, E149 |
| Diabetes with complications | 250 | E102, E103, E104, E105, E112, E113, E114, E115, E117, E122, E123, E124, E125, E127, E132, E133, E134, E135, E137, E142, E143, E144, E145, E147 |
| Paraplegia and hemiplegia | 334, 342, 343, 344 | G81, G82, G041, G114, G801, G802, G830, G831, G932, G833, G834, G839 |
| Renal disease | 403, 582, 583, 585, 586, 588, V56 | N18, N19, N052, N053, N054, N055, N056, N057, N250, I120, I131, N032, N033, N034, N035, N036, N037, Z490, Z491, Z492, Z940, Z992 |
| Cancer | 140, 141, 142, 143, 144, 145, 146, 147, 148, 149, 150, 151, 152, 153, 154, 155, 156, 157, 158, 159, 160, 161, 162, 163, 164, 165, 170, 171, 172, 174, 175, 176, 179, 180, 181, 182, 183, 184, 185, 168, 187, 188, 189, 190, 191, 192, 193, 194, 195, 200, 201, 202, 203, 204, 205, 206, 207, 208, 238 | C00, C01,C02, C03, C04, C05, C06, C07, C08, C09, C10, C11, C12, C13, C14, C15, C16, C17, C18, C19, C20, C21, C22, C23, C24, C25, C26, C30, C31, C32, C33, C34, C37, C38, C39, C40, C41, C43, C45, C46, C47, C48, C49, C50, C51, C52, C53, C54, C55 C56, C57, C58, C60, C61, C62, C63, C64, C65, C66, C67, C68, C69, C70, C71, C72, C73, C74, C75, C76, C81, C82, C83, C84, C85, C88, C90, C91, C92, C93, C94, C95, C96,C97 |
| Metastatic Carcinoma | 196, 197, 198, 199 | C77, C78. C79. C80 |

**Table 2.** Number of healthcare related visits (HRV) per individual in each calendar year by hepatitis C (HCV) co-infection status.

1. People living with HIV

| ART Initiation Year | Number of individuals | Number of individuals without a visit, N (%) | Healthcare-related Visits | | |
| --- | --- | --- | --- | --- | --- |
|  |  |  | Median  number per  individual | Mean  number per  individual | Rate per person-year |
|  |  |  | (Q1-Q3) | (Range, SD) |  |
| 2000 | 87 | <5 (4.6) | 15 (8-25) | 17 (0-48; 12) | 34 |
| 2001 | 173 | 6 (3.5) | 19 (11-28) | 21 (0-81; 15) | 28 |
| 2002 | 268 | 15 (5.6) | 17 (10-27) | 19 (0-75; 14) | 25 |
| 2003 | 395 | 13 (3.3) | 16 (10-28) | 21 (0-204; 19) | 25 |
| 2004 | 513 | 17 (3.3) | 16 (10-28) | 22 (0-217; 20) | 25 |
| 2005 | 657 | 27 (4.1) | 16 (10-27) | 21 (0-164; 20) | 24 |
| 2006 | 804 | 23 (2.9) | 16 (9-25) | 20 (0-280; 18) | 22 |
| 2007 | 970 | 33 (3.4) | 14 (9-23) | 19 (0-175; 17) | 21 |
| 2008 | 1198 | 36 (3) | 14 (9-23) | 19 (0-302; 18) | 21 |
| 2009 | 1445 | 39 (2.7) | 14 (9-22) | 18 (0-307; 18) | 20 |
| 2010 | 1656 | 53 (3.2) | 13 (8-21) | 18 (0-310; 20) | 20 |
| 2011 | 1889 | 55 (2.9) | 13 (8-21) | 18 (0-319; 20) | 19 |
| 2012 | 2091 | 52 (2.5) | 13 (8-21) | 18 (0-298; 20) | 19 |
| 2013 | 2097 | 45 (2.1) | 12 (8-19) | 17 (0-294; 18) | 18 |

1. People living with HIV/HCV

| ART Initiation Year | Number of individuals | Number of individuals without a visit, N (%) | Healthcare-related Visits | | |
| --- | --- | --- | --- | --- | --- |
|  |  |  | Median  number per  individual | Mean  number per individual | Rate per person-year |
|  |  |  | (Q1-Q3) | (Range, SD) |  |
| 2000 | 98 | 7 (7.1) | 17 (6-36) | 28 (0-125; 32) | 49 |
| 2001 | 190 | 10 (5.3) | 19 (6-41.75) | 33 (0-176; 39) | 45 |
| 2002 | 268 | 15 (5.6) | 20 (11-41.25) | 37 (0-230; 43) | 45 |
| 2003 | 375 | 15 (4) | 20 (9.5-49.5) | 36 (0-310; 41) | 43 |
| 2004 | 482 | 14 (2.9) | 22 (11.25-48) | 38 (0-277; 41) | 43 |
| 2005 | 576 | 23 (4) | 21 (10-49.25) | 38 (0-301; 43) | 44 |
| 2006 | 663 | 23 (3.5) | 21 (11-44) | 36 (0-249; 41) | 41 |
| 2007 | 806 | 28 (3.5) | 20 (10-41) | 34 (0-315; 38) | 39 |
| 2008 | 927 | 28 (3) | 21 (11-45.5) | 35 (0-388; 40) | 39 |
| 2009 | 1056 | 36 (3.4) | 21 (11-42) | 34 (0-418; 38) | 37 |
| 2010 | 1166 | 36 (3.1) | 21 (11-43) | 33 (0-373; 38) | 36 |
| 2011 | 1270 | 44 (3.5) | 20 (10-43) | 32 (0-551; 36) | 35 |
| 2012 | 1303 | 25 (1.9) | 20 (10.5-44) | 32 (0-386; 34) | 34 |
| 2013 | 1284 | 22 (1.7) | 20 (10-44) | 31 (0-520; 33) | 34 |

**Notes:** ART: combination antiretroviral therapy; Q1-Q3: 25^th^ - 75^th^ percentiles; Range: minimum to maximum of the data, SD: standard deviation

**REFERENCES**

1. British Columbia Centre for Disease Control. HIV/AIDS Information System (HAISYS). Clinical Prevention Services, British Columbia Centre for Disease Control, 2016. <http://www.bccdc.ca/about/accountability/data-access-requests/public-health-data>.

2. British Columbia Centre for Disease Control Public Health Laboratory. HIV laboratory testing datasets (tests: ELISA, Western blot, NAAT, p24, culture). Clinical Prevention Services, British Columbia Centre for Disease Control, 2016. <http://www.bccdc.ca/about/accountability/data-access-requests/public-health-data>.

3. Patterson S, Cescon A, Samji H, Cui Z, Yip B, Lepik KJ, et al. Cohort Profile: HAART Observational Medical Evaluation and Research (HOMER) cohort. Int J Epidemiol 2015;44:58-67.

4. British Columbia Ministry of Health (2016). Medical Services Plan (MSP) Payment Information File; Consolidation File (MSP Registration & Premium Billing); Home & Community Care (Continuing Care); Mental Health; PharmaNet. British Columbia Ministry of Health. Data Extract. MOH (2016). <http://www2.gov.bc.ca/gov/content/health/conducting-health-research-evaluation/data-access-health-data-central>.

5. Canadian Institute of Health Information (2016). Discharge Abstract Database (Hospital Separations). British Columbia Ministry of Health. Data Extract. MOH (2016). <http://www2.gov.bc.ca/gov/content/health/conducting-health-research-evaluation/data-access-health-data-central>.

6. British Columbia Vital Statistics Agency (2016). Vital Statistics Deaths. British Columbia Ministry of Health. Data Extract. MOH (2016). <http://www2.gov.bc.ca/gov/content/health/conducting-health-research-evaluation/data-access-health-data-central>.

7. Quan H, Li B, Couris CM, Fushimi K, Graham P, Hider P, et al. Updating and validating the Charlson comorbidity index and score for risk adjustment in hospital discharge abstracts using data from 6 countries. Am J Epidemiol. 2011;173:676-682
